# Topical bromfenac reduces multiple inflammatory cytokines in the aqueous humour of pseudophakic patients

**DOI:** 10.1038/s41598-021-85495-w

**Published:** 2021-03-16

**Authors:** Takehiro Matsumura, Kentaro Iwasaki, Shogo Arimura, Ryuji Takeda, Yoshihiro Takamura, Masaru Inatani

**Affiliations:** 1 Department of Ophthalmology, Faculty of Medical Sciences, University of Fukui, 23-3 Shimoaizuki, Matsuoka, Eiheiji, Fukui 910-1193 Japan; 2 Department of Ophthalmology, Tokyo Dental College Ichikawa General Hospital, Chiba, Japan; 3 Department of Nutritional Sciences for Well-Being, Faculty of Health Sciences for Welfare, Kansai University of Welfare Sciences, Osaka, Japan

**Keywords:** Eye diseases, Glaucoma

## Abstract

Intraocular surgery is associated with increased ocular inflammation. If maintained for a prolonged period after surgery, this inflammation can cause various complications, including subconjunctival fibrosis and bleb scarring. This clinical trial was a prospective, randomised, single-blind, interventional study comparing the efficacy and safety of 0.1% bromfenac sodium ophthalmic solution and 0.02% fluorometholone ophthalmic suspension in the inhibition of multiple inflammatory cytokines in the aqueous humour of 26 patients with pseudophakic eyes who had undergone phacoemulsification and intraocular lens implantation. The patients were randomly assigned to one of the trial drugs, and aqueous humour samples were collected before and after drug administration. Platelet-derived growth factor-AA levels significantly decreased in both drug groups, but they were significantly higher in the fluorometholone group than in the bromfenac group (*P* = 0.034). Bromfenac also significantly decreased vascular endothelial growth factor level (*P* = 0.0077), as well as monocyte chemoattractant protein-1 level (*P* = 0.013), which was elevated for a prolonged period after phacoemulsification. These data suggest that bromfenac is useful to alleviate prolonged microenvironmental alterations in the aqueous humour of pseudophakic eyes.

## Introduction

Intraocular surgery causes the breakdown of the blood–aqueous barrier, together with ocular inflammation^1–6^. These events elevate the levels of proteins, cytokines, and growth factors in the aqueous humour. The levels of several inflammatory cytokines, such as monocyte chemoattractant protein (MCP)-1 and interleukin (IL)-8, remain elevated in the aqueous humour over 1 year after phacoemulsification, despite the recent small-incision surgery^7,8^. Elevated MCP-1 level is significantly associated with a poor surgical outcome after trabeculectomy in glaucomatous eyes^7^. Phacoemulsification causes prolonged alterations in the aqueous humour microenvironment, which promotes subconjunctival fibrosis and bleb scarring after trabeculectomy in pseudophakic eyes^7,8^. Therefore, inhibiting these inflammatory cytokines may prevent bleb scarring after trabeculectomy in these patients. Long-term administration of inflammatory cytokine-inhibiting eye drops postoperatively may be a potential therapeutic strategy to improve surgical outcomes in pseudophakic eyes with poor prognoses compared with those in phakic eyes^9,10^.

Steroidal and non-steroidal anti-inflammatory drug (NSAID)-based drops are candidates for anti-inflammatory eye drops; however, long-term administration of steroid eye drops increase the risk of intraocular pressure (IOP) elevation and ocular infection. Therefore, we focused on NSAID eye drops, which have no risk of IOP elevation and ocular infection as with steroidal eye drops. However, unlike topical steroids, there are no reports on whether NSAID eye drops reduce inflammatory cytokine levels in pseudophakic eyes.

In this prospective study, we investigated the inhibitory effects of an NSAID eye drop formulation, 0.1% bromfenac sodium ophthalmic solution^11,12^, on the inflammatory cytokine levels in pseudophakic eyes following phacoemulsification.

## Methods

### Study design

This clinical trial was a prospective, randomised, single-blind (investigators and assessors were blinded), interventional study comparing the efficacy and safety of 0.1% bromfenac sodium ophthalmic solution and 0.02% fluorometholone ophthalmic suspension in the inhibition of inflammatory cytokines in the aqueous humour of pseudophakic eyes. The study protocol was approved by the Institutional Review Board of University of Fukui Hospital, Fukui, Japan (approval number: 20148028). Written informed consent was obtained from all patients after a detailed explanation of the study-specific procedures involved. The study was conducted in accordance with the tenets of the Declaration of Helsinki. This study was registered at the University Hospital Medical Information Network Clinical Trials Registry (UMIN) of Japan (ID: UMIN000017934; date of access and registration: 24 August 2015).

### Patient selection

**Figure 1:**
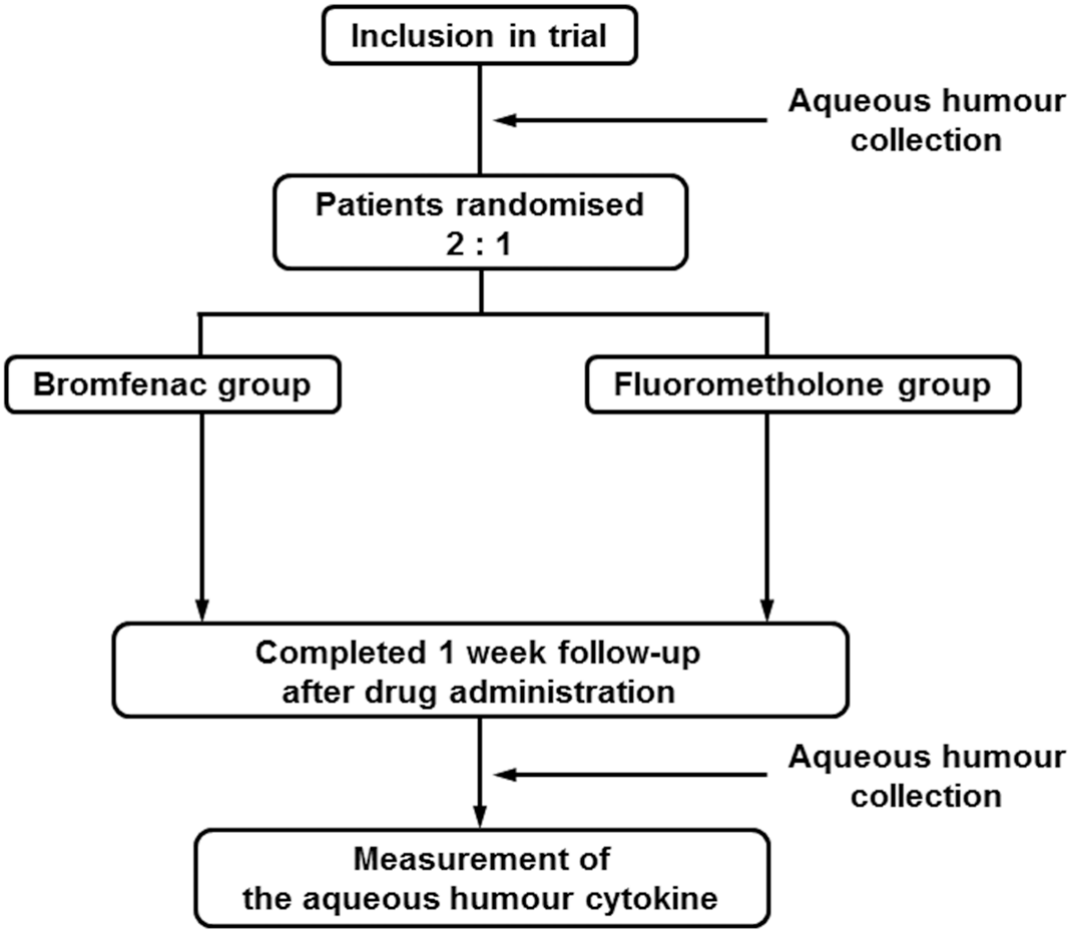
Study design. Patients with pseudophakic eyes who met the inclusion criteria were randomised 2:1 to receive either bromfenac or fluorometholone eye drops. All investigators and statisticians were blinded to the randomisation of the patients. Both trial eye drops were administered twice daily for 1 week. Aqueous humour was collected from patients before and 1 week after trial drug administration. Cytokine levels in the aqueous humour were measured using multiplex immunoassay, and the changes before and after drug administration were analysed.

Patients with pseudophakic eyes who were previously treated with phacoemulsification and intraocular lens (IOL) implantation were eligible for this study. The enrolled patients were screened based on the following inclusion criteria: (1) age, 20 years or older; (2) a pseudophakic eye; and (3) phacoemulsification and IOL implantation more than 3 months before the study. The exclusion criteria were as follows: (1) a history of hypersensitivity to bromfenac and fluorometholone; (2) active ocular inflammation or infection in either eye (including allergic conjunctivitis and vernal conjunctivitis); (3) a history of diabetic retinopathy; (4) glaucoma; (5) ophthalmic surgery (including laser treatment) within the past 3 months; (6) systemic diseases that might affect the efficacy or safety of the trial eye drops; (7) treatment with systemic corticosteroids, systemic immunosuppression, topical corticosteroids, or prostaglandin analogues; and (8) being ruled ineligible for any other reason by the doctor in charge. The participants were enrolled between 1 January 2016 and 31 October 2018 at University of Fukui Hospital. One eye from each patient was included in the study. The number of cases was set according to a previous report^7^. A 0.02% fluorometholone ophthalmic suspension was used as the control drug, and patients were randomised in a 2:1 allocation ratio to receive 0.1% bromfenac sodium ophthalmic solution or 0.02% fluorometholone ophthalmic suspension in the study eye (Fig. 1). Bromfenac has been approved for use twice a day and fluorometholone 2–4 times a day; therefore, to align the number of drops for research, both drugs were administered twice daily for 1 week. Allocation was concealed until the completion of cytokine measurements in all patients. The investigators were blinded to the randomisation of the patients, although the patients were not blinded to the trial drugs. During the administration period, the use steroid or NSAID eye drops and oral medications other than the study drugs was prohibited.

### Sample collection and cytokine measurements

Aqueous humour was collected from the patients before and 1 week after trial drug administration. At least 100 μL of sample was obtained as gently as possible using a 30-gauge needle under sterile conditions, taking care not to touch the iris, IOL, or corneal endothelium to prevent contamination with blood. The collected aqueous humour was immediately frozen at − 80 °C and stored until analysis. The samples were anonymised and sent to a clinical testing company (LSI Medience Corporation, Tokyo, Japan) to measure the concentrations of MCP-1, IL-6, IL-8, tumour necrosis factor (TNF)-α, platelet-derived growth factor (PDGF)-AA, and vascular endothelial growth factor (VEGF) as described previously^13^, using a MILLIPLEX _MAP_ Human Cytokine/Chemokine Magnetic Bead Panel kit (Merck Millipore, Billerica, MA, USA) according to the manufacturer’s instructions. Briefly, colour-coded microspheres with two fluorescent dyes coated with specific capture antibodies were allowed to react with 25 μL of each sample or with a standard solution at 5 °C overnight. The incubated beads were washed twice; subsequently, a biotinylated detection antibody was introduced and incubated at 20–25 °C for 1 h. Streptavidin–phycoerythrin, which binds to the biotinylated detection antibody, was used to analyse the reaction mixture. The levels of MCP-1, IL-6, IL-8, TNF-α, PDGF-AA, and VEGF were measured using a Bio-Plex Suspension Array System (Bio-Rad Laboratories, Hercules, CA, USA). Sample concentrations were calculated automatically by Bio-Plex Manager Software (Bio-Rad Laboratories) using a standard curve of known concentrations of recombinant human protein standards (supplied with the kits).

### Outcome measures

The primary outcome for this study was to compare the differences in the cytokine levels in the aqueous humour before and 1 week after the administration of the trial drugs. The secondary outcome was to compare the cytokine levels before and 1 week after drug administration in each drug group.

We also recorded other patient characteristics, including age, sex, the presence or absence of ocular disease and systemic disease, and the period after phacoemulsification. A stratified analysis was performed on the relationship between inflammatory cytokine levels in the aqueous humour and patient characteristics, such as age, sex, the presence or absence of hypertension or type 2 diabetes mellitus, and the period after phacoemulsification.

To assess the safety of the trial drug, ophthalmic data before and after drug administration were analysed, including IOP, best-corrected visual acuity (BCVA), corneal and conjunctival findings, anterior chamber inflammation, and fundus findings. These aspects were examined by slit-lamp microscopy and binocular ophthalmoscopy. We evaluated corneal disorder using the National Eye Institute-recommended grading system^14^.

### Statistical analyses

Statistical comparisons of patient characteristics between the bromfenac and fluorometholone groups were performed using Mann–Whitney *U* nonparametric test and Fisher’s exact test. The changes in cytokine concentrations before and after drug administration in each drug group were analysed using paired *t* test. The differences in cytokine concentrations, IOP, and BCVA before and after drug administration between the trial drug groups were analysed using Student’s *t*-test. The BCVA values were recorded as decimal acuities and were converted to the logarithm of the minimum angle of resolution (logMAR) for statistical analyses. Fisher’s exact test was used to compare the ocular findings before and after drug administration. Mann–Whitney *U* nonparametric test and Fisher’s exact test were used to compare relationships between inflammatory cytokine levels in the aqueous humour and patient characteristics. Statistical analyses were performed by a statistician (R.T.). Data are presented as mean ± standard deviation (SD), and results with *P* < 0.05 were considered statistically significant.

## Results

### Patient characteristics

We enrolled 26 patients (i.e., 26 eyes) in this study. Of these, 17 and 9 were randomised to the bromfenac and fluorometholone groups, respectively. The baseline characteristics of the patients are summarised in Table 1. One patient withdrew from the study at the time of aqueous humour collection 1 week after drug administration. There were no significant differences with respect to age, sex, the presence or absence of systemic and ocular diseases, period after phacoemulsification, baseline IOP, or BCVA between the bromfenac and fluorometholone groups.

**Table 1:** Patient demographic data.

Characteristic	0.1% Bromfenac	0.02% Fluorometholone	*P* value
Number of eyes, *n*	17	9	
**Age (years)**			
Mean ± SD	81.5 ± 7.3	76.4 ± 9.7	0.23
Range	65–96	64–91	
Sex (male/female)	6/11	4/5	0.69
**Presence of systemic diseases**			
Diabetes mellitus (+ / −)	4/13	2/7	1.00
Hypertension (+ / −)	11/6	6/3	1.00
**Presence of ocular diseases**			
Dry eye, *n* (%)	1 (5.9)	0 (0)	1.00
Epiretinal membrane, *n* (%)	0 (0)	1 (11)	0.35
**Period after phacoemulsification (months)**			
Mean ± SD	48.9 ± 54.5	27.4 ± 31.9	0.29
Range	3–189	3–86	
IOP (mean ± SD, mmHg)	12.9 ± 3.2	12.2 ± 3.6	0.61
BCVA (logMAR units ± SD)	0.14 ± 0.16	0.21 ± 0.20	0.35

Statistical comparisons between the bromfenac and fluorometholone groups were performed using Mann–Whitney *U* and Fisher’s exact tests.

*SD* standard deviation, *IOP* intraocular pressure, *BCVA* best-corrected visual acuity, *logMAR* logarithm of the minimum angle of resolution.

### Cytokine concentration outcomes

The five cytokines (IL-6, IL-8, MCP-1, PDGF-AA, and VEGF) analysed were detectable in the aqueous humour of patients with pseudophakic eyes before and after drug administration. In many patients, the level of TNF-α was less than the lower limit of quantification before and after trial drug administration; therefore, the effect of the drug on this cytokine could not be determined.

**Figure 2:**
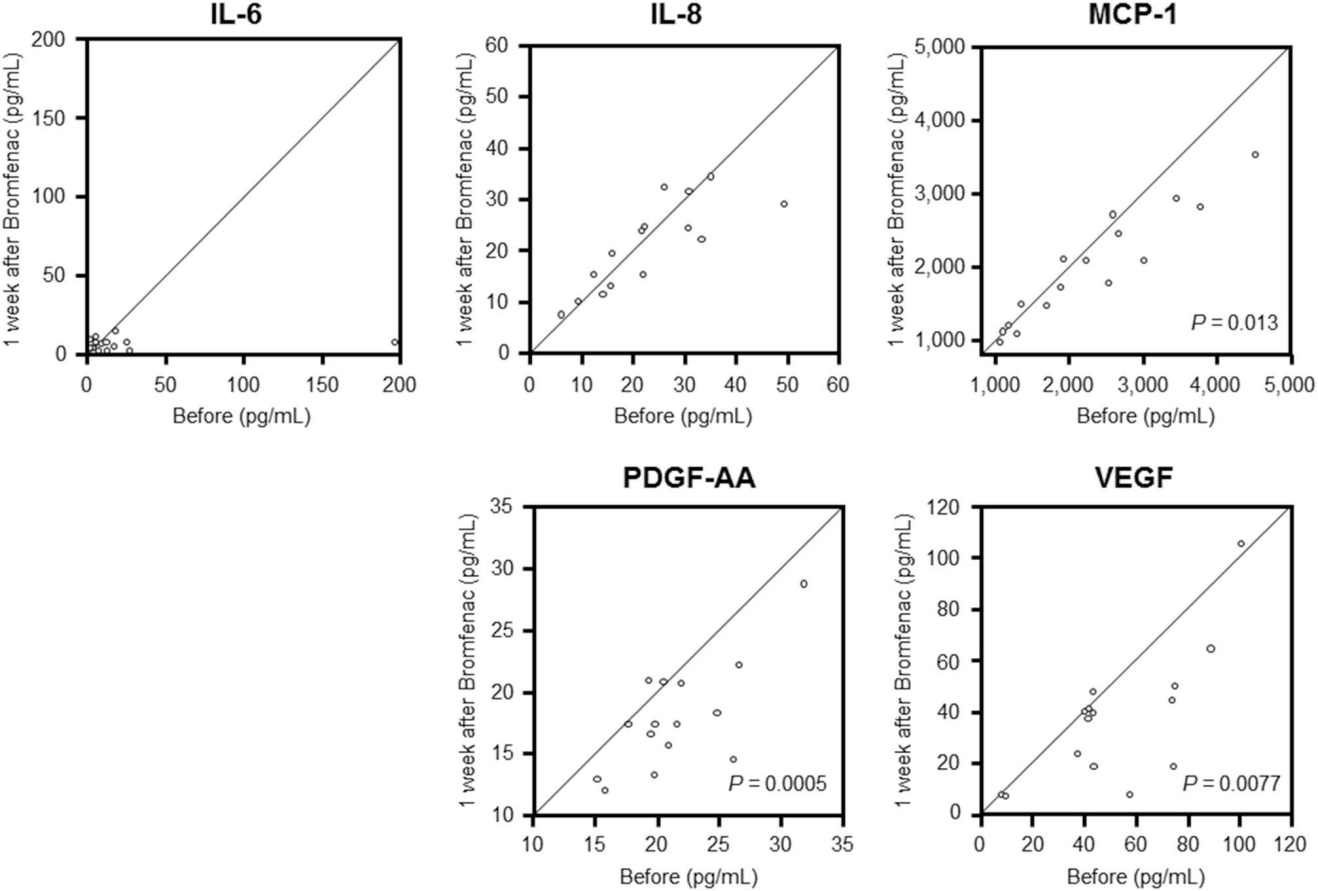
Scattergrams of inflammatory cytokine concentrations in the aqueous humour of patients with pseudophakic eyes before and after bromfenac administration. The x- and y-axes indicate the levels of each cytokine before and after treatment, respectively. The concentrations of MCP-1, PDGF-AA, and VEGF in the aqueous humour significantly decreased 1 week after bromfenac administration (*P* = 0.013, *P* = 0.0005, and *P* = 0.0077, respectively, paired *t* test). There were no significant changes in the concentrations of IL-6 or IL-8 before and 1 week after bromfenac administration. *IL* interleukin, *MCP-1* monocyte chemoattractant protein-1, *PDGF-AA* platelet-derived growth factor-AA, *VEGF* vascular endothelial growth factor.

In the bromfenac group, the mean ± SD concentrations of IL-6, IL-8, MCP-1, PDGF-AA, and VEGF in the aqueous humour before drug administration were 21.17 ± 46.64, 22.11 ± 11.07, 2275.94 ± 1002.13, 21.04 ± 4.67, and 47.95 ± 29.37 pg/mL, respectively. After drug administration, their concentrations were 6.64 ± 3.50, 21.07 ± 8.59, 1975.21 ± 757.16, 17.95 ± 4.31, and 35.16 ± 26.69 pg/mL, respectively. The concentrations of MCP-1, PDGF-AA, and VEGF significantly decreased 1 week after bromfenac administration (*P* = 0.013, *P* = 0.0005, and *P* = 0.0077, respectively; Fig. 2 and Table 2). There were no significant changes in the concentrations of IL-6 and IL-8 before and 1 week after bromfenac administration.

**Table 2:** Changes in cytokine levels after drug administration.

Drug	Cytokine		Before (pg/mL)	After (pg/mL)	Change (pg/mL)	*P* value (each group)	*P* value (between groups) Before	*P* value (between groups) After	*P* value (between groups) Change
Bromfenac	IL-6	Mean	21.17	6.64	− 16.14	0.23	0.025	0.082	0.67
		± SD	46.64	3.50	49.52				
	IL-8	Mean	22.11	21.07	− 1.82	0.32	0.068	0.14	0.17
		± SD	11.07	8.59	6.77				
	MCP-1	Mean	2275.94	1975.21	− 286.42	0.013	0.0054	0.025	0.090
		± SD	1002.13	757.16	406.75				
	PDGF-AA	Mean	21.04	17.95	− 3.42	0.0005	0.026	0.20	0.034
		± SD	4.67	4.31	3.23				
	VEGF	Mean	47.95	35.16	− 13.98	0.0077	0.80	0.51	0.39
		± SD	29.37	26.69	19.61				
Fluorometholone	IL-6	Mean	176.68	124.27	− 52.41	0.66	–	–	–
		± SD	265.50	254.78	320.62				
	IL-8	Mean	82.48	32.52	− 49.96	0.29	–	–	–
		± SD	131.96	26.88	132.89				
	MCP-1	Mean	4538.35	3287.40	− 1250.94	0.12	–	–	–
		± SD	2763.88	1973.91	2141.40				
	PDGF-AA	Mean	29.25	21.46	− 7.78	0.0058	–	–	–
		± SD	12.94	8.77	6.25				
	VEGF	Mean	51.26	28.16	− 23.10	0.056	–	–	–
		± SD	34.81	21.35	31.04				

The paired *t* test was used to analyse the changes in cytokine levels before and after the administration of each drug. The differences in cytokine concentrations before and after drug administration between the drug groups were analysed using Student’s *t* test. Results with *P* < 0.05 were considered statistically significant.

*SD* standard deviation, *IL* interleukin, *MCP-1* monocyte chemoattractant protein-1, *PDGF-AA* platelet-derived growth factor-AA, *VEGF* vascular endothelial growth factor.

**Figure 3:**
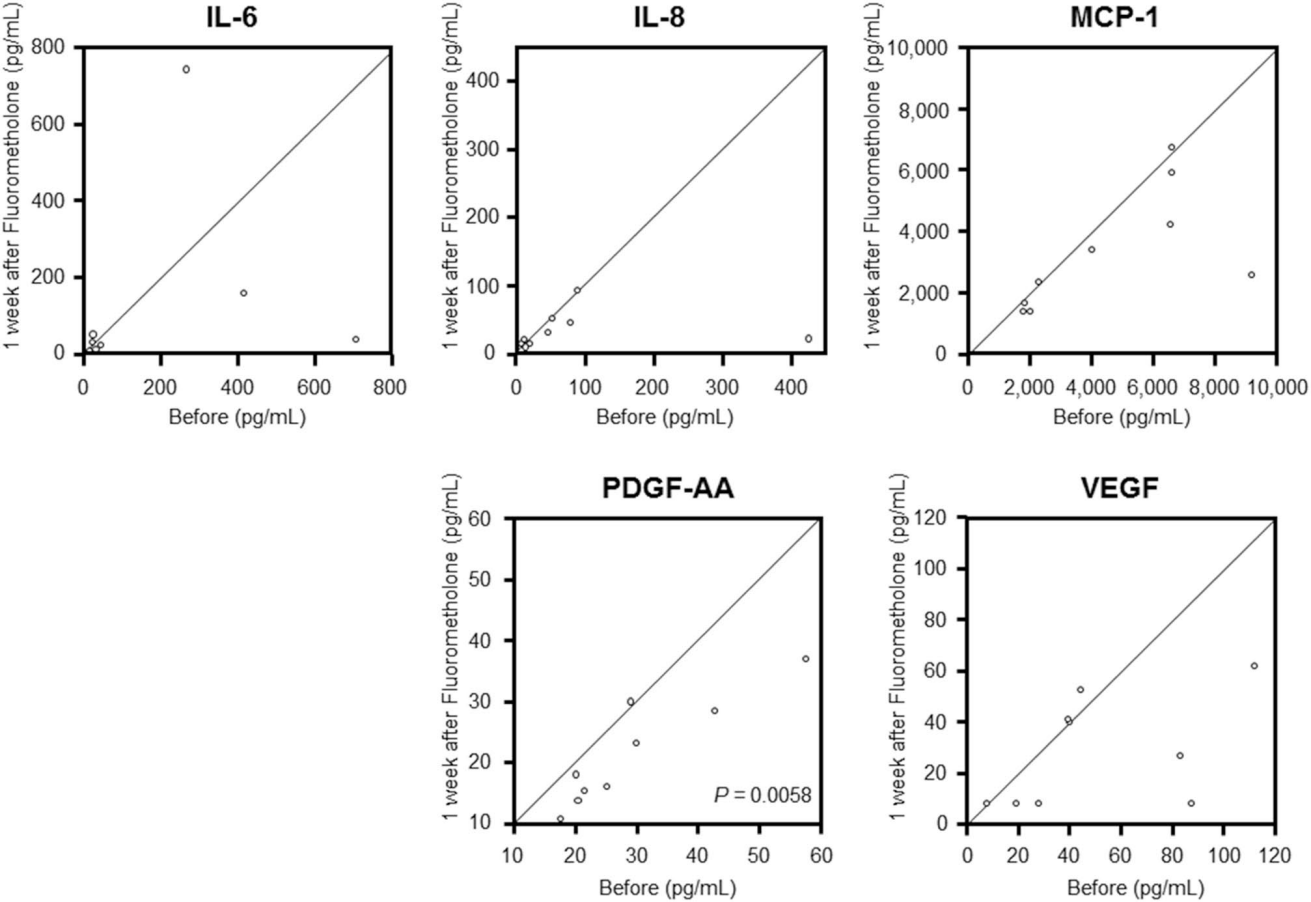
Scattergrams of inflammatory cytokine concentrations in the aqueous humour of patients with pseudophakic eyes before and after fluorometholone administration. The x- and y-axes indicate the levels of each cytokine before and after treatment, respectively. The concentrations of PDGF-AA in the aqueous humour significantly decreased 1 week after fluorometholone administration (*P* = 0.0058, paired *t* test). There were no significant changes in the concentrations of IL-6, IL-8, MCP-1, or VEGF before and 1 week after fluorometholone administration. *IL* interleukin, *MCP-1* monocyte chemoattractant protein-1, *PDGF-AA* platelet-derived growth factor-AA, *VEGF* vascular endothelial growth factor.

In the fluorometholone group, the mean ± SD concentrations of IL-6, IL-8, MCP-1, PDGF-AA, and VEGF in the aqueous humour before drug administration were 176.68 ± 265.50, 82.48 ± 131.96, 4538.35 ± 2763.88, 29.25 ± 12.94, and 51.26 ± 34.81 pg/mL, respectively. After drug administration, their concentrations were 124.27 ± 254.78, 32.52 ± 26.88, 3287.40 ± 1973.91, 21.46 ± 8.77, and 28.16 ± 21.35 pg/mL, respectively. The PDGF-AA concentration was significantly decreased 1 week after fluorometholone administration (*P* = 0.0058; Fig. 3 and Table 2). There were no significant changes in the concentrations of IL-6, IL-8, MCP-1, and VEGF before and 1 week after fluorometholone administration.

The change in the PDGF-AA concentration before and after drug administration was significantly larger in the fluorometholone group than in the bromfenac group (*P* = 0.034). The other cytokines did not show any significant differences between the bromfenac and fluorometholone groups with respect to their concentration changes before and after drug administration (Table 2).

Next, we evaluated the statistical relationships between the inflammatory cytokine levels and different patient characteristics, such as age, sex, presence or absence of hypertension or type 2 diabetes mellitus, or the time elapsed since phacoemulsification. There were no significant relationships between the concentrations of any inflammatory cytokine and the patient characteristics before the administration of the drugs. The results of the stratified analyses of the relationships between inflammatory cytokine concentrations and patient characteristics after the administration of each drug are shown in Supplementary Tables S1–S5.

### Safety

No serious adverse events were observed in this study, and there were no abnormal findings in the cornea, conjunctiva, or anterior chamber before and after drug administration. One patient in the bromfenac group exhibited dry eye disease, which was treated with purified sodium hyaluronate instillation during the study period. No abnormal corneal findings were observed by slit-lamp microscopy. One patient exhibited an epiretinal membrane; however, no change was observed in the ocular fundus before and after drug administration.

In the bromfenac group, the mean ± SD IOP values before and after drug administration were 12.9 ± 3.2 and 12.3 ± 4.1 mmHg, respectively; in the fluorometholone group, these values were 12.2 ± 3.6 and 12.3 ± 3.0 mmHg, respectively. There were no significant differences with respect to the changes in the IOP values before and after drug administration.

In the bromfenac group, the mean ± SD BCVA values before and after drug administration were 0.14 ± 0.16 and 0.14 ± 0.15 logMAR units, respectively; in the fluorometholone group, these values were 0.21 ± 0.20 and 0.18 ± 0.18 logMAR units, respectively. There were no significant differences with respect to the changes in the BCVA values before and after drug administration.

## Discussion

The primary outcome of this study was to compare the effects of bromfenac and fluorometholone on changes in cytokine levels in the aqueous humour 1 week after drug administration compared to before administration in pseudophakic eyes following phacoemulsification. After drug administration, the PDGF-AA concentration in the aqueous humour significantly decreased in both groups; in particular, the reduction rate was significantly higher in the fluorometholone group than in the bromfenac group. In addition, the bromfenac group showed a significant decrease in the concentrations of MCP-1 and VEGF in the aqueous humour after drug administration. However, there were no significant changes in the concentrations of IL-6 or IL-8 before and 1 week after the administration of the two drugs.

Previous studies indicate that inflammatory cytokines such as MCP-1 and IL-8 remain elevated in the aqueous humour for a long period (more than 1 year) after phacoemulsification, which affects subconjunctival fibrosis and bleb scarring after trabeculectomy in pseudophakic eyes^7,8,10,15^. However, it is not known whether NSAID eye drops reduce such inflammatory cytokines in the aqueous humour of patients with pseudophakic eyes. The results of the present study clearly demonstrate that bromfenac decreases the concentrations of MCP-1, PDGF-AA, and VEGF in the aqueous humour of pseudophakic eyes following phacoemulsification.

MCP-1 recruits and activates inflammatory cells, which are important for wound healing^16–18^ and are a key factor in bleb scarring after trabeculectomy in pseudophakic eyes^7,8,10^. In the present study, the MCP-1 concentration in the aqueous humour of pseudophakic eyes was comparable with or higher than previously reported levels^7^, although an average of 42 months had elapsed since phacoemulsification. In addition, there was no significant relationship between the period after phacoemulsification and the MCP-1 concentration in the aqueous humour. These findings indicate that phacoemulsification affects the microenvironment of the aqueous humour for several years after surgery. Postoperative inflammation is not detectable by slit-lamp microscopic examination or laser flare photometry several months after phacoemulsification^19^. However, subclinical or mild inflammation with cytokine elevation in the anterior chamber can last for long periods. Chronic inflammation is associated with subconjunctival fibrosis and bleb scarring after trabeculectomy^7,8^. Bromfenac eye drops have been used as an acute postoperative anti-inflammatory treatment^20^, and our results suggest that they could also be effective against MCP-1-induced chronic inflammation by reducing the MCP-1 level, which might improve the prognosis of trabeculectomy in pseudophakic eyes. Kawai et al. reported that the mean concentration of MCP-1 in the aqueous humour before phacoemulsification was 796.9 pg/mL, and it increased by more than 120% after surgery^7^. In this study, we observed a decrease of only 13.21% (from 2275.94 to 1975.21 pg/mL) in the mean concentration of MCP-1 in the eyes treated with bromfenac; therefore, future studies should investigate if the magnitude of this effect has any clinical relevance.

In contrast to the effect of bromfenac in the present study, eye drops containing ketorolac tromethamine, another NSAID, significantly reduced the IL-8 and PDGF-AA levels in the eyes with proliferative diabetic retinopathy^21^. Bromfenac decreased the aqueous concentrations of MCP-1, PDGF-AA, and VEGF; therefore, the type of NSAID may influence which cytokines are reduced. The significant decreases in PDGF-AA and VEGF concentrations after bromfenac administration are interesting because increases in these cytokines are associated with various intraocular diseases, such as open angle glaucoma^8^, diabetic retinopathy^22,23^, retinal vein occlusion^24,25^, and age-related macular degeneration^25,26^. However, the inhibitory changes induced by bromfenac were small, because PDGF-AA and VEGF do not increase after phacoemulsification^7^. Therefore, it is unclear whether bromfenac is clinically effective in controlling these cytokines in other ocular diseases. Further investigations will be needed to assess this.

There were no adverse events associated with the use of bromfenac eye drops in this study, although this may be due to the short-term administration period. Topical NSAIDs are commonly used for several months following phacoemulsification, to treat acute postoperative inflammation and prevent cystoid macular oedema^27^. The most serious complications associated with topical NSAIDs are corneal disorders, including corneal erosion, ulceration, and melting^28^. Therefore, the long-term use of NSAID eye drops requires monitoring for the development of corneal disorders and may be inappropriate if there are complications with the ocular surface. However, bromfenac leads to a lower frequency of corneal disorders than another NSAID, diclofenac sodium ophthalmic solution^28^, and may be safer and easier to use for other ocular conditions, such as after trabeculectomy. Further prospective comparative studies are needed to assess these issues.

The present study had some limitations. First, we did not compare bromfenac with other NSAIDs or with stronger steroids such as dexamethasone, which have been reported to have greater inhibitory effects on the inflammatory cytokine cascade. However, bromfenac has been reported to be more effective than dexamethasone and fluorometholone in preventing cystoid macular oedema following phacoemulsification^29–31^. We chose fluorometholone as a comparison control drug because stronger steroid eye drops have strong side effects that would conflict with the aim of this study and the ethics of the research. Therefore, we could not conclude whether bromfenac is the most effective anti-inflammatory agent for attenuating the elevated levels of inflammatory cytokines in the aqueous humour of pseudophakic eyes. Second, fluorometholone may also have the potential to inhibit multiple inflammatory cytokines; however, its effects are unclear because of the small sample size of the fluorometholone group. Although we did not obtain aqueous humour samples from a large number of patients for ethical reasons, a larger sample size would aid in determining the effects of fluorometholone.

In conclusion, our results provide direct clinical evidence that topical 0.1% bromfenac sodium ophthalmic solution significantly reduces the levels of MCP-1, PDGF-AA, and VEGF in the aqueous humour of patients with pseudophakic eyes after phacoemulsification. Our data suggest that bromfenac might be useful for reducing prolonged alterations in the aqueous humour microenvironment in pseudophakic eyes; however, the clinical relevance of these findings is yet to be determined.

## Supplementary Information

Supplementary Information.

## Data Availability

All data generated or analysed during this study are included in this published article (and its Supplementary Information files).
