# Supplementary material for: Topical bromfenac reduces multiple inflammatory cytokines in the aqueous humour of pseudophakic patients
Source: Sci Rep. 2021 Mar 16;11:6018. doi: 10.1038/s41598-021-85495-w (PMC7966778; doi:10.1038/s41598-021-85495-w)
Supplement: Supplementary file 1 — Supplementary Information. [file 41598_2021_85495_MOESM1_ESM.pdf]

**Topical Bromfenac Reduces Multiple Inflammatory Cytokines in the Aqueous Humour of Pseudophakic Patients**

Takehiro Matsumura[1,2], Kentaro Iwasaki[1], Shogo Arimura[1], Ryuji Takeda[3], Yoshihiro Takamura[1], Masaru Inatani[1,*]

[1]Department of Ophthalmology, Faculty of Medical Sciences, University of Fukui, Fukui, Japan
[2]Department of Ophthalmology, Tokyo Dental College Ichikawa General Hospital, Chiba, Japan
[3]Department of Nutritional Sciences for Well-Being, Faculty of Health Sciences for Welfare, Kansai University of Welfare Sciences, Osaka, Japan

[*]Correspondence and requests for materials should be addressed to:
Masaru Inatani,
Department of Ophthalmology, Faculty of Medical Sciences, University of Fukui, 23-3 Shimoaizuki, Matsuoka, Eiheiji, Fukui 910-1193, Japan
Phone: +81-776-61-8403; Fax: +81-776-61-8131; E-mail: inatani@u-fukui.ac.jp

| MCP-1 | | Drug | | Before (pg/mL) | After (pg/mL) | Change (pg/mL) | *P* value (each group) | *P* value (between groups) Before | *P* value (between groups) After | *P* value (between groups) Change |
|---|---|---|---|---|---|---|---|---|---|---|
| **Sex** | Male | Bromfenac | Mean | 1667.46 | 1447.15 | −52.83 | 0.44 | 0.020 | 0.056 | 0.18 |
| | | | ± SD | 587.66 | 418.78 | 137.51 | | | | |
| | | Fluorometholone | Mean | 5412.40 | 3371.37 | −2041.03 | 0.28 | | | |
| | | | ± SD | 3193.62 | 1848.83 | 3065.07 | | | | |
| | Female | Bromfenac | Mean | 2607.83 | 2215.23 | −392.60 | 0.016 | 0.18 | 0.20 | 0.53 |
| | | | ± SD | 1044.43 | 766.16 | 448.24 | | | | |
| | | Fluorometholone | Mean | 3839.10 | 3220.23 | −618.87 | 0.24 | | | |
| | | | ± SD | 2500.70 | 2283.93 | 1000.79 | | | | |
| **Diabetes mellitus** | + | Bromfenac | Mean | 2166.81 | 2096.81 | 42.69 | 0.67 | 0.0032 | 0.20 | 0.19 |
| | | | ± SD | 565.67 | 614.82 | 149.60 | | | | |
| | | Fluorometholone | Mean | 7900.97 | 4242.78 | −3658.20 | 0.43 | | | |
| | | | ± SD | 1849.78 | 2352.48 | 4202.26 | | | | |
| | − | Bromfenac | Mean | 2309.51 | 1947.15 | −362.37 | 0.0081 | 0.091 | 0.10 | 0.47 |
| | | | ± SD | 1119.75 | 805.65 | 412.00 | | | | |
| | | Fluorometholone | Mean | 3577.60 | 3014.44 | −563.16 | 0.12 | | | |
| | | | ± SD | 2183.83 | 1970.17 | 829.13 | | | | |
| **Hypertension** | + | Bromfenac | Mean | 2277.84 | 1985.87 | −269.27 | 0.067 | 0.012 | 0.081 | 0.061 |
| | | | ± SD | 1065.18 | 857.86 | 408.71 | | | | |
| | | Fluorometholone | Mean | 5032.69 | 3183.19 | −1849.50 | 0.12 | | | |
| | | | ± SD | 2921.84 | 1715.63 | 2453.00 | | | | |

| | | | | | | | | | | |
|---|---|---|---|---|---|---|---|---|---|---|
| | – | Bromfenac | Mean | 2272.44 | 1957.43 | −315.01 | 0.14 | 0.31 | 0.22 | 0.39 |
| | | | ± SD | 971.82 | 628.18 | 440.54 | | | | |
| | | Fluorometholone | Mean | 3549.65 | 3495.83 | −53.82 | 0.77 | | | |
| | | | ± SD | 2648.36 | 2851.17 | 273.75 | | | | |
| **Period after phacoemulsification** | < 3 years | Bromfenac | Mean | 2506.90 | 2190.28 | −316.91 | 0.13 | 0.0007 | 0.013 | 0.20 |
| | | | ± SD | 920.48 | 671.25 | 476.03 | | | | |
| | | Fluorometholone | Mean | 6603.73 | 4661.44 | −1942.29 | 0.31 | | | |
| | | | ± SD | 2118.34 | 1980.32 | 3147.48 | | | | |
| | ≥ 3 years | Bromfenac | Mean | 2292.11 | 1885.36 | −406.76 | 0.042 | 0.46 | 0.55 | 0.40 |
| | | | ± SD | 1266.32 | 969.92 | 366.47 | | | | |
| | | Fluorometholone | Mean | 3150.69 | 2339.56 | −811.13 | 0.22 | | | |
| | | | ± SD | 2275.65 | 1335.91 | 1043.57 | | | | |

**Supplementary Table S1. Changes in the MCP-1 levels after drug administration**

The stratified analysis was performed on the relationship between the MCP-1 levels in the aqueous humour and patient characteristics. The paired *t*-test was used to analyse the changes in the MCP-1 levels before and after the administration of each drug. The differences in the MCP-1 concentration before and after drug administration between the drug groups were analysed using Student *t*-test. Results with $P < 0.05$ were considered statistically significant.

MCP-1, monocyte chemoattractant protein-1; SD, standard deviation.

| PDGF-AA | | Drug | | Before (pg/mL) | After (pg/mL) | Change (pg/mL) | *P* value (each group) | *P* value (between groups) Before | *P* value (between groups) After | *P* value (between groups) Change |
|---|---|---|---|---|---|---|---|---|---|---|
| Sex | Male | Bromfenac | Mean | 20.15 | 16.41 | −2.90 | 0.020 | 0.029 | 0.069 | 0.025 |
| | | | ± SD | 4.71 | 4.08 | 1.72 | | | | |
| | | Fluorometholone | Mean | 37.77 | 26.05 | −11.72 | 0.04 | | | |
| | | | ± SD | 15.64 | 9.08 | 6.75 | | | | |
| | Female | Bromfenac | Mean | 21.53 | 18.72 | −3.69 | 0.014 | 0.73 | 0.76 | 0.66 |
| | | | ± SD | 4.80 | 4.42 | 3.83 | | | | |
| | | Fluorometholone | Mean | 22.43 | 17.79 | −4.64 | 0.062 | | | |
| | | | ± SD | 4.54 | 7.34 | 4.02 | | | | |
| Diabetes mellitus | + | Bromfenac | Mean | 19.05 | 13.55 | −3.71 | 0.053 | 0.15 | 0.22 | 0.18 |
| | | | ± SD | 4.40 | 1.85 | 1.54 | | | | |
| | | Fluorometholone | Mean | 39.38 | 26.22 | −13.16 | 0.32 | | | |
| | | | ± SD | 25.36 | 15.26 | 10.10 | | | | |
| | − | Bromfenac | Mean | 21.66 | 19.05 | −3.35 | 0.0079 | 0.13 | 0.69 | 0.15 |
| | | | ± SD | 4.74 | 4.05 | 3.58 | | | | |
| | | Fluorometholone | Mean | 26.35 | 20.10 | −6.25 | 0.013 | | | |
| | | | ± SD | 8.49 | 7.34 | 4.77 | | | | |
| Hypertension | + | Bromfenac | Mean | 21.43 | 19.23 | −2.84 | 0.011 | 0.048 | 0.34 | 0.020 |
| | | | ± SD | 4.93 | 4.55 | 2.57 | | | | |
| | | Fluorometholone | Mean | 31.93 | 22.67 | −9.26 | 0.019 | | | |
| | | | ± SD | 15.12 | 8.88 | 6.62 | | | | |

| | | | | | | | | | | |
|---|---|---|---|---|---|---|---|---|---|---|
| | – | Bromfenac | Mean | 20.33 | 16.04 | −4.29 | 0.052 | 0.34 | 0.50 | 0.87 |
| | | | ± SD | 4.50 | 3.39 | 4.14 | | | | |
| | | Fluorometholone | Mean | 23.87 | 19.05 | −4.82 | 0.25 | | | |
| | | | ± SD | 5.78 | 9.86 | 5.20 | | | | |
| **Period after phacoemulsification** | < 3 years | Bromfenac | Mean | 20.39 | 17.53 | −3.47 | 0.012 | 0.02 | 0.061 | 0.14 |
| | | | ± SD | 6.33 | 6.01 | 2.22 | | | | |
| | | Fluorometholone | Mean | 37.55 | 27.76 | −9.80 | 0.13 | | | |
| | | | ± SD | 15.79 | 9.01 | 9.29 | | | | |
| | ≥ 3 years | Bromfenac | Mean | 21.60 | 19.19 | −2.40 | 0.021 | 0.67 | 0.031 | 0.035 |
| | | | ± SD | 2.58 | 2.32 | 1.77 | | | | |
| | | Fluorometholone | Mean | 20.79 | 14.74 | −6.06 | 0.024 | | | |
| | | | ± SD | 3.11 | 3.10 | 2.85 | | | | |

**Supplementary Table S2. Changes in the PDGF-AA levels after drug administration**

The stratified analysis was performed on the relationship between the PDGF-AA levels in the aqueous humour and patient characteristics. The paired *t*-test was used to analyse the changes in the PDGF-AA levels before and after the administration of each drug. The differences in the PDGF-AA concentration before and after drug administration between the drug groups were analysed using Student *t*-test. Results with $P < 0.05$ were considered statistically significant.

PDGF-AA, platelet-derived growth factor-AA; SD, standard deviation.

| VEGF | | Drug | | Before (pg/mL) | After (pg/mL) | Change (pg/mL) | *P* value (each group) | *P* value (between groups) Before | *P* value (between groups) After | *P* value (between groups) Change |
|---|---|---|---|---|---|---|---|---|---|---|
| **Sex** | Male | Bromfenac | Mean | 40.17 | 27.67 | −6.54 | 0.30 | 0.82 | 0.98 | 0.58 |
| | | | ± SD | 28.88 | 20.74 | 12.16 | | | | |
| | | Fluorometholone | Mean | 44.84 | 27.21 | −17.63 | 0.45 | | | |
| | | | ± SD | 32.48 | 22.79 | 41.17 | | | | |
| | Female | Bromfenac | Mean | 52.19 | 38.90 | −17.71 | 0.032 | 0.82 | 0.52 | 0.45 |
| | | | ± SD | 30.11 | 29.51 | 22.05 | | | | |
| | | Fluorometholone | Mean | 56.40 | 28.92 | −27.48 | 0.066 | | | |
| | | | ± SD | 39.47 | 22.80 | 24.53 | | | | |
| **Diabetes mellitus** | + | Bromfenac | Mean | 38.81 | 23.96 | −4.46 | 0.42 | 0.79 | 0.27 | 0.33 |
| | | | ± SD | 25.32 | 16.00 | 7.72 | | | | |
| | | Fluorometholone | Mean | 47.54 | 8.00 | −39.54 | 0.50 | | | |
| | | | ± SD | 55.92 | 0.00 | 55.92 | | | | |
| | − | Bromfenac | Mean | 50.76 | 37.96 | −16.37 | 0.021 | 0.92 | 0.75 | 0.85 |
| | | | ± SD | 30.87 | 28.60 | 21.16 | | | | |
| | | Fluorometholone | Mean | 52.32 | 33.92 | −18.40 | 0.10 | | | |
| | | | ± SD | 32.99 | 20.82 | 25.45 | | | | |
| **Hypertension** | + | Bromfenac | Mean | 47.57 | 39.21 | −10.28 | 0.15 | 0.40 | 0.68 | 0.21 |
| | | | ± SD | 31.06 | 30.71 | 19.26 | | | | |
| | | Fluorometholone | Mean | 62.35 | 32.91 | −29.45 | 0.11 | | | |
| | | | ± SD | 38.10 | 22.61 | 36.83 | | | | |

| | | | | | | | | | | |
|---|---|---|---|---|---|---|---|---|---|---|
| | − | Bromfenac | Mean | 48.63 | 29.08 | −19.54 | 0.067 | 0.30 | 0.48 | 0.50 |
| | | | ± SD | 28.79 | 20.31 | 20.52 | | | | |
| | | Fluorometholone | Mean | 29.08 | 18.67 | −10.41 | 0.21 | | | |
| | | | ± SD | 10.44 | 18.48 | 10.04 | | | | |
| **Period after phacoemulsification** | < 3 years | Bromfenac | Mean | 36.22 | 29.81 | −5.50 | 0.29 | 0.63 | 0.60 | 0.42 |
| | | | ± SD | 20.22 | 15.36 | 11.30 | | | | |
| | | Fluorometholone | Mean | 43.77 | 24.00 | −19.77 | 0.39 | | | |
| | | | ± SD | 32.58 | 18.48 | 39.54 | | | | |
| | ≥ 3 years | Bromfenac | Mean | 53.35 | 46.06 | −7.28 | 0.24 | 0.80 | 0.37 | 0.040 |
| | | | ± SD | 40.57 | 36.94 | 13.32 | | | | |
| | | Fluorometholone | Mean | 60.50 | 26.15 | −34.35 | 0.053 | | | |
| | | | ± SD | 44.32 | 25.34 | 22.08 | | | | |

**Supplementary Table S3. Changes in the VEGF levels after drug administration**

The stratified analysis was performed on the relationship between the VEGF levels in the aqueous humour and patient characteristics. The paired *t*-test was used to analyse the changes in the VEGF levels before and after the administration of each drug. The differences in the VEGF concentration before and after drug administration between the drug groups were analysed using Student *t*-test. Results with $P < 0.05$ were considered statistically significant.

VEGF, vascular endothelial growth factor; SD, standard deviation.

| **IL-6** | | **Drug** | | **Before (pg/mL)** | **After (pg/mL)** | **Change (pg/mL)** | ***P* value (each group)** | ***P* value (between groups) Before** | ***P* value (between groups) After** | ***P* value (between groups) Change** |
|---|---|---|---|---|---|---|---|---|---|---|
| **Sex** | Male | Bromfenac | Mean | 11.09 | 5.40 | −6.81 | 0.34 | 0.079 | 0.19 | 0.15 |
| | | | ± SD | 11.77 | 3.12 | 13.98 | | | | |
| | | Fluorometholone | Mean | 283.86 | 51.79 | −232.07 | 0.24 | | | |
| | | | ± SD | 342.05 | 71.95 | 314.62 | | | | |
| | Female | Bromfenac | Mean | 26.66 | 7.26 | −20.81 | 0.30 | 0.38 | 0.10 | 0.075 |
| | | | ± SD | 57.59 | 3.67 | 60.46 | | | | |
| | | Fluorometholone | Mean | 69.50 | 196.76 | 127.26 | 0.36 | | | |
| | | | ± SD | 129.82 | 363.69 | 234.09 | | | | |
| **Diabetes mellitus** | + | Bromfenac | Mean | 4.56 | 5.76 | 1.49 | 0.47 | 0.0034 | 0.12 | 0.055 |
| | | | ± SD | 1.98 | 2.82 | 2.90 | | | | |
| | | Fluorometholone | Mean | 561.57 | 98.39 | −463.18 | 0.26 | | | |
| | | | ± SD | 206.06 | 82.58 | 288.63 | | | | |
| | − | Bromfenac | Mean | 26.27 | 6.86 | −20.55 | 0.22 | 0.54 | 0.15 | 0.091 |
| | | | ± SD | 52.71 | 3.73 | 54.89 | | | | |
| | | Fluorometholone | Mean | 48.38 | 132.90 | 84.52 | 0.33 | | | |
| | | | ± SD | 105.78 | 298.59 | 193.04 | | | | |
| **Hypertension** | + | Bromfenac | Mean | 29.62 | 7.73 | −26.47 | 0.24 | 0.058 | 0.13 | 0.13 |
| | | | ± SD | 56.98 | 3.62 | 62.98 | | | | |
| | | Fluorometholone | Mean | 228.24 | 42.35 | −185.89 | 0.23 | | | |
| | | | ± SD | 321.27 | 65.79 | 291.38 | | | | |

| | | | | | | | | | | |
|---|---|---|---|---|---|---|---|---|---|---|
| | − | Bromfenac | Mean | 5.66 | 5.01 | −0.66 | 0.81 | 0.18 | 0.15 | 0.13 |
| | | | ± SD | 4.42 | 2.83 | 6.32 | | | | |
| | | Fluorometholone | Mean | 90.74 | 260.81 | 170.07 | 0.38 | | | |
| | | | ± SD | 150.24 | 416.88 | 266.84 | | | | |
| **Period after phacoemulsification** | < 3 years | Bromfenac | Mean | 6.48 | 5.77 | 0.13 | 0.95 | 0.0056 | 0.13 | 0.57 |
| | | | ± SD | 4.29 | 3.04 | 6.49 | | | | |
| | | Fluorometholone | Mean | 349.51 | 236.84 | −112.67 | 0.67 | | | |
| | | | ± SD | 291.24 | 342.61 | 479.47 | | | | |
| | ≥ 3 years | Bromfenac | Mean | 14.76 | 8.21 | −6.56 | 0.24 | 0.14 | 0.40 | 0.15 |
| | | | ± SD | 10.21 | 4.30 | 12.31 | | | | |
| | | Fluorometholone | Mean | 4.59 | 15.07 | 10.48 | 0.45 | | | |
| | | | ± SD | 1.01 | 19.01 | 19.50 | | | | |

**Supplementary Table S4. Changes in the IL-6 levels after drug administration**

The stratified analysis was performed on the relationship between the IL-6 levels in the aqueous humour and patient characteristics. The paired *t*-test was used to analyse the changes in the IL-6 levels before and after the administration of each drug. The differences in the IL-6 concentration before and after drug administration between the drug groups were analysed using Student *t*-test. Results with $P < 0.05$ were considered statistically significant.

IL, interleukin; SD, standard deviation.

| IL-8 | | Drug | | Before (pg/mL) | After (pg/mL) | Change (pg/mL) | *P* value (each group) | *P* value (between groups) Before | *P* value (between groups) After | *P* value (between groups) Change |
|---|---|---|---|---|---|---|---|---|---|---|
| Sex | Male | Bromfenac | Mean | 11.53 | 12.87 | 1.39 | 0.27 | 0.12 | 0.14 | 0.27 |
| | | | ± SD | 3.47 | 4.69 | 2.46 | | | | |
| | | Fluorometholone | Mean | 142.54 | 39.56 | −102.98 | 0.38 | | | |
| | | | ± SD | 191.20 | 35.96 | 200.14 | | | | |
| | Female | Bromfenac | Mean | 27.88 | 25.17 | −3.43 | 0.20 | 0.51 | 0.80 | 0.49 |
| | | | ± SD | 9.29 | 7.00 | 7.75 | | | | |
| | | Fluorometholone | Mean | 34.44 | 26.90 | −7.54 | 0.32 | | | |
| | | | ± SD | 30.29 | 19.66 | 14.70 | | | | |
| Diabetes mellitus | + | Bromfenac | Mean | 13.89 | 16.79 | 2.21 | 0.093 | 0.078 | 0.23 | 0.28 |
| | | | ± SD | 5.66 | 7.37 | 1.26 | | | | |
| | | Fluorometholone | Mean | 256.84 | 57.29 | −199.56 | 0.51 | | | |
| | | | ± SD | 238.21 | 49.39 | 287.60 | | | | |
| | − | Bromfenac | Mean | 24.64 | 22.14 | −2.83 | 0.20 | 0.36 | 0.58 | 0.35 |
| | | | ± SD | 11.22 | 8.82 | 7.25 | | | | |
| | | Fluorometholone | Mean | 32.66 | 25.45 | −7.21 | 0.19 | | | |
| | | | ± SD | 27.05 | 17.16 | 13.02 | | | | |
| Hypertension | + | Bromfenac | Mean | 22.21 | 22.08 | −1.44 | 0.60 | 0.073 | 0.19 | 0.19 |
| | | | ± SD | 11.77 | 9.28 | 7.87 | | | | |
| | | Fluorometholone | Mean | 111.38 | 36.55 | −74.84 | 0.31 | | | |
| | | | ± SD | 156.89 | 29.71 | 161.33 | | | | |

| | | | | | | | | | | |
|---|---|---|---|---|---|---|---|---|---|---|
| | – | Bromfenac | Mean | 21.94 | 19.55 | −2.39 | 0.32 | 0.81 | 0.64 | 0.52 |
| | | | ± SD | 10.71 | 8.03 | 5.35 | | | | |
| | | Fluorometholone | Mean | 24.67 | 24.47 | −0.20 | 0.83 | | | |
| | | | ± SD | 24.45 | 23.22 | 1.46 | | | | |
| **Period after phacoemulsification** | < 3 years | Bromfenac | Mean | 19.51 | 20.17 | −0.42 | 0.78 | 0.054 | 0.058 | 0.23 |
| | | | ± SD | 9.40 | 9.36 | 3.46 | | | | |
| | | Fluorometholone | Mean | 153.26 | 49.31 | −103.96 | 0.37 | | | |
| | | | ± SD | 182.28 | 31.05 | 199.47 | | | | |
| | ≥ 3 years | Bromfenac | Mean | 26.12 | 20.93 | −5.20 | 0.22 | 0.81 | 0.99 | 0.65 |
| | | | ± SD | 15.05 | 8.91 | 9.15 | | | | |
| | | Fluorometholone | Mean | 29.82 | 20.81 | −9.01 | 0.36 | | | |
| | | | ± SD | 32.88 | 16.37 | 16.54 | | | | |

**Supplementary Table S5. Changes in the IL-8 levels after drug administration**

The stratified analysis was performed on the relationship between the IL-8 levels in the aqueous humour and patient characteristics. The paired *t*-test was used to analyse the changes in the IL-8 levels before and after the administration of each drug. The differences in the IL-8 concentration before and after drug administration between the drug groups were analysed using Student *t*-test. Results with $P < 0.05$ were considered statistically significant.

IL, interleukin; SD, standard deviation.
